# The Lipopolysaccharide Export Pathway in *Escherichia coli*: Structure, Organization and Regulated Assembly of the Lpt Machinery

**DOI:** 10.3390/md12021023

**Published:** 2014-02-17

**Authors:** Alessandra Polissi, Paola Sperandeo

**Affiliations:** Department of Biotechnology and Biosciences, University of Milano-Bicocca, Piazza della Scienza 2, 20126 Milan, Italy; E-Mail: paola.sperandeo@unimib.it

**Keywords:** outer membrane, lipopolysaccharide transport, *Escherichia coli*

## Abstract

The bacterial outer membrane (OM) is a peculiar biological structure with a unique composition that contributes significantly to the fitness of Gram-negative bacteria in hostile environments. OM components are all synthesized in the cytosol and must, then, be transported efficiently across three compartments to the cell surface. Lipopolysaccharide (LPS) is a unique glycolipid that paves the outer leaflet of the OM. Transport of this complex molecule poses several problems to the cells due to its amphipatic nature. In this review, the multiprotein machinery devoted to LPS transport to the OM is discussed together with the challenges associated with this process and the solutions that cells have evolved to address the problem of LPS biogenesis.

## 1. Introduction

All living cells are surrounded by the cytoplasmic membrane, a unit membrane of which overall architecture, a fluid lipid bilayer with integral and peripheral membrane proteins, is conserved among the three domains of life *Bacteria*, *Archaea*, and *Eukarya*. The importance of such a structure is underlined by the notion that everything that exists outside of the biological membrane is non-living.

Outside of the universally conserved cytoplasmic membrane, prokaryotes have developed complex architectures, collectively named the cell wall, which provide additional strength, contribute to the cell shape determination, and allow them to face unpredictable and often hostile environmental insults [1,2,3]. Many *Bacteria* are surrounded by an additional lipid bilayer, the outer membrane (OM), and are, thus, described as diderm bacteria. The OM is not present in monoderm bacteria, which possess the cytoplasmic membrane as the unique lipid membrane [4,5,6].

The OM is an essential structure that has been extensively studied over the last half century in *Proteobacteria* phylum and particularly in *Enterobacteriaceae*, it possesses a peculiar asymmetric structure due to the presence of a complex glycolipid, the lipopolysaccharide (LPS), in the outer leaflet of the lipid bilayer, whereas the inner leaflet is composed of phospholipids. OM proteins (OMPs) and lipoproteins are also embedded and anchored, respectively, in the OM [7]. The space in between IM and OM is an aqueous compartment, the periplasm, which contains a peptidoglycan layer, a large cell polymer that surrounds the bacterial cell [8,9]. This cellular exoskeleton is the main structure responsible for the cell shape and the mechanical strength and elasticity of the bacterial envelope [10]. The presence of a highly structured OM poses several problems for its biogenesis. Both lipid and protein components must not only be synthesized in the cytoplasm and/or at the inner membrane (IM) level and translocated across the IM lipid bilayer, but must also traverse the aqueous periplasmic space and be assembled at the amphypathic final destination. The cell compartments external to the IM are devoid of ATP and other high-energy carriers [11]. As a consequence the energy to build up OM structures is either provided by exergonic reactions (thus involving substrates that have been energized before their translocation across the IM) or transduced by devices (usually protein machines) connected to the IM and capable to exploit the energy released by ATP hydrolysis in the cytoplasm or the proton motive force.

This review, after a short overview on the OM structure and functions will focus on its peculiar component, the LPS. First LPS structure and biosynthetic pathway will be briefly discussed. Then, the machinery that ferries LPS from the IM to its final destination in the OM together with recent advances in the molecular mechanisms of LPS export will be reviewed in more details.

## 2. Overview of OM Structure and Functions

Several structural and functional aspects differentiate the OM from the cytoplasmic membrane (IM). The most striking structural difference is the asymmetry of the OM bilayer as the periplasmic side is made by a layer of the same type of phospholipids that compose both leaflets of the IM, whereas LPS constitutes the OM layer facing the environment outside the cell. The unique chemical structure and properties of LPS, as discussed in below, are mainly responsible of the peculiar properties of the OM. Indeed, the presence of this peculiar glycolipid in the outer leaflet of the OM, endows Gram-negative bacteria with a strong permeability barrier, which accounts for the generally higher resistance of Gram-negative bacteria, as compared to most Gram-positives, to many toxic chemicals such as antibiotics and detergents (e.g., bile salts), thus, allowing survival in hostile environments (e.g., gastrointestinal tracts of mammals) encountered during host colonization or infection [12,13].

### 2.1. OM Proteins and Lipoproteins

Cell-environment exchanges across the OM are ensured by OM proteins (OMPs) implicated in several functions, such as nutrients uptake, transport, and secretion of various molecules (proteins, polysaccharides, drugs). While integral IM proteins are typically composed of α-helical bundles [14] the vast majority of OMPs are integral proteins that assume a β-barrel topology [15]. OM-associated proteins are generally lipoproteins that are anchored to the periplasmic side of the OM via a lipid tail attached to an *N*-terminal cysteine residue [16]. In *Escherichia* *coli* the majority of lipoproteins are OM associated, and only a minor fraction faces the periplasm by IM anchoring. The most abundant lipoprotein in *E. coli* is Lpp (or Braun’s protein) that anchors the peptidoglycan layer to the OM [17].

OMPs and lipoproteins are synthesised as pre-proteins in the cytoplasm and then secreted across the IM by the SEC translocase a universally conserved machine that transport unfolded proteins [18].

Following translocation, lipoproteins are processed into mature forms on the periplasmic side of cytoplasmic membrane where a lipid moiety is attached to the N terminus to anchor these proteins to the membrane. [16]. Lipoprotein sorting has been extensively studied in *E. coli* where their traffic system is essential for growth [19,20]. Indeed in *E. coli* lipoproteins play vital roles in OM sorting of OMPs, LPS and lipoprotein themselves [3]. Lipoproteins with OM location are transported by the dedicated Lol system (LolABCDE) composed by five essential proteins. LolCDE constitute an ABC transporter that initiates sorting by mediating the detachment of lipoproteins from the IM and transferring them to the periplasmic chaperone LolA. The water-soluble lipoprotein-LolA complex crosses the periplasm and at the OM LolA transfers its cargo to LolB for incorporation into the lipid bilayer [20].

Once secreted across the IM, misfolding of β-barrel OM proteins precursors in the periplasm is prevented by molecular chaperones, such as SurA and Skp [21], which assists OMPs transport across the periplasm and deliver them to the BAM complex a molecular machine driving β-barrel assembly [22]. The Bam machinery is composed by the essential BamA OMP that work in complex and is tightly associated with four lipoproteins partners BamB, BamC, BamD, and BamE. The Bam complex may be viewed as a modular molecular machine in which BamA forms the protein:lipid interface at which OMP substrates enter into the lipid phase of the membrane. BamB interacts with BamA and is proposed to form a scaffold to assist β-barrel folding. BamB, BamC, and BamD interact and form a module suggested to drive a conformational switch in the BAM complex that enables β-barrel insertion into the OM [22].

### 2.2. LPS Structure and Function

**Figure 1:**
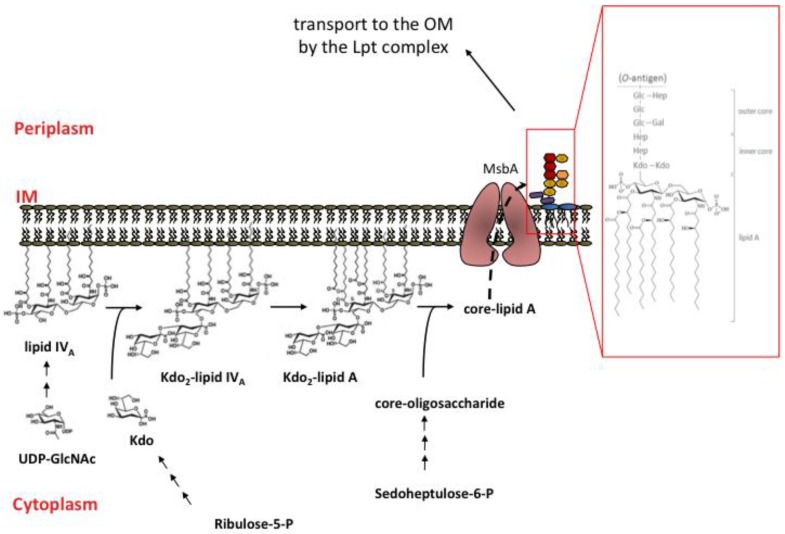
LPS biosynthesis in *E. coli* K12 strains. Cytoplasm and inner leaflet of IM: lipid IV_A_ is synthesized from two molecules of the sugar nucleotide UDP-GlcNAc. Sequential addition of two Kdo molecules to lipid IV_A_ produces the Kdo_2_-lipid IV_A_ moiety which undergoes two late acylation reactions to give Kdo_2_-lipid A. Core oligosaccharide is assembled on Kdo_2_-lipid A via sequential glycosyl transfer from nucleotide sugar precursors. Outer leaflet of IM: core-lipid A is translocated across the IM by MsbA transporter. Schematic representation of core lipid A: violet rectangle, Kdo; yellow heptagon, heptose; red hexagon, glucose; orange hexagon, galactose. In the inset the chemical structure of *E. coli* K12 LPS is shown.

LPS is typically organized into three structural domains: lipid A, a core oligosaccharide and a highly variable *O*-antigen constituted of repeating oligosaccharide units (Figure 1) [23]. The core is covalently linked to lipid A and can be further divided into inner (lipid A proximal) and outer core. The chemical structure of the outer core is variable, whereas the inner core tends to be quite conserved and in all species so far analyzed, it contains at least one residue of 3-deoxy-d-*manno*-oct-2 ulosonic acid (Kdo) linking the inner core to lipid A. Therefore, Kdo is a chemical hallmark of LPS and a marker of LPS-producing Gram-negative bacteria [24]. The *O*-antigen is the distal, surface exposed LPS moiety responsible of the immunogenic properties of this macromolecule [23].

LPS is essential in most Gram-negative bacteria with the notable exception of *Neisseria meningitidis* [25]. The LPS structural requirements for bacterial viability however, may vary across genera/species. In *E. coli* the minimal LPS structure required for growth has been defined as Kdo_2_-lipid A [23], although the lethal phenotype of Kdo-deficient mutants may be rescued by several suppressor mutations [26]. In contrast, *Pseudomonas aeruginosa*, in addition to lipid A, requires the full inner core and at least part of the outer core to be viable [27,28]. The structural complexity of LPS reflects the multiple functions displayed by this macromolecule. While the outer hydrophilic layer of LPS leaflet in the OM represents an effective barrier for passive diffusion of lipophilic compounds, the lipid A moiety, together with the phospholipids of the internal leaflet, forms a hydrophobic barrier. Moreover, LPS appears tightly packed at the outer OM leaflet as strong lateral interaction between LPS molecules occurs by the bridging action of Mg^2+^ and Ca^2+^ divalent cations, thus, counteracting the negative repulsive charges and stabilizing the structure [12,24].

LPS is also a potent activator of the innate immune response and lipid A (also known as endotoxin) represents the conserved pathogen associated molecular pattern (PAMP) recognized by innate immune receptors to signal and activate complex signaling cascades that lead to the release of pro-inflammatory cytokines [29]. Recognition of lipid A requires the TLR4-MD2 complex [30,31] and the accessory protein CD14 and LPB (LPS binding protein) [32].

### 2.3. Overview of LPS Biosynthesis

The biosynthesis of LPS is a complex process that occurs in three different cellular compartments, cytoplasm, IM and periplasm, thus, requiring spatial and temporal coordination of several independent biosynthetic pathways (lipid A, Kdo, and oligosaccharide core) that converge in an ordered assembly line to give the mature molecule (Figure 1) [23,33,34]. Nine enzymes are required to for the synthesis of the Kdo_2_-lipid A moiety. The first reaction in the synthesis of lipid A is the fatty acylation of UDP-*N*-acetyglucosamine (UDP-GlcNAc) by LpxA to UDP-3-*O*-acyl-GlcNAc that is deacetylated by the LpxC enzyme [35]. Further acylation is then carried out by LpxD the third enzyme of the biosynthetic machinery. In both acylation steps catalyzed by LpxA and LpxD the fatty acyl donor is R-3–hydroxymyristoyl ACP that also serves as an acyl donor for the synthesis of membrane phospholipids and therefore represents a crucial metabolic branch point in the biosynthesis of membrane components [36,37]. Next, reactions catalyzed by LpxH, LpxB, and LpxK result in the synthesis of the tetra-acylated lipid IV_A_ that, in *E. coli*, is the substrate of WaaA the CMP-Kdo dependent transferase that catalyzes the sequential incorporation of two Kdo residues synthesized in a separate pathway [23]. Finally, the late acyltransferases LpxL and LpxM catalyze the addition of secondary acyl chains to the distal glucosamine [38,39] to give the hexa-acylated Kdo_2_-lipid A moiety. The additional sugars composing the oligosaccharide core are added to Kdo_2_-lipid A by specific glycosyl-transferases to generate the core-lipid A structure.

The core-lipid A, which is anchored to the IM with its hydrophilic moiety exposed to the cytoplasm, is then flipped over the IM by the ABC transporter MsbA and becomes exposed in the periplasm [40,41]. *O*-antigen repeat units are synthesized in the cytoplasm and then flipped at the periplasmic face of the IM attached to the lipid carrier undecaprenyl diphosphate. Mature LPS is then formed at the periplasmic side of the IM by the WaaL ligase that catalyzes the ligation of *O*-antigen to core-lipid A [42]. The *O*-antigen moiety is not essential and is missing in common laboratory *E. coli* K12 strains due to inactivation of the *wbbL* gene [43,44] but is present in most wild type strains and clinical isolates where it contributes to virulence by protecting the bacteria from phagocytosis and complement-mediated killing [45].

The enzymes for lipid A and Kdo biosynthesis are constitutively expressed [23,46]. However, in *E. coli* the production of Kdo_2_-lipid A is post-transcriptionally regulated by FtsH, an essential membrane protease belonging to the AAA family (ATPase associated with various cellular activities) that controls the turnover of LpxC [47] the enzyme that catalyzes the first committed step of the lipid A biosynthetic pathway [48]. Mutations in *ftsH* lead to increased cellular levels of LpxC and are lethal [47]. This can be explained by the fact that both lipid A and phospholipid biosynthetic pathways largely depend on the same precursor molecule, R-3–hydroxymyristoyl ACP. The increased level of LpxC may, thus, effectively deplete the R-3–hydroxymyristoyl ACP pool, thus, leading to an imbalanced phospholipid/LPS ratio in the OM [47]. FtsH also controls the turnover of WaaA, the enzyme catalyzing incorporation of Kdo residues in lipid IV_A_ [49]. Therefore FtsH dependent proteolysis seems to be a crucial point of control to balance the levels of membrane lipid synthesis according to the physiological state/growth conditions of the cell. Indeed it has been recently reported that LpxC is degraded rapidly during slow growth, presumably to avoid toxic overproduction of LPS, and that the alarmone ppGpp is needed together with FtsH to control LpxC stability [50]. Very recently an additional player of this regulatory mechanism, YciM a conserved tetratricopeptide repeat protein, has been identified. YciM is essential and its depletion results in increased LpxC levels leading to higher amount of LPS and, ultimately, to cell death suggesting that FtsH proteolytic activity is also modulated by YciM [51]. Control of LPS biosynthesis by FtsH mediated proteolysis, however, is not a widespread mechanism across Gram-negative bacteria but seems restricted to *Enterobacteria*; indeed the *C*-terminus of LpxC (where sequence specific degradation signals are located) differs significantly among species whereas the overall sequence of LpxC is highly conserved [52].

## 3. LPS transport to the Outer Membrane

### 3.1. The Lpt Machinery: Structure and Organization of the Components across IM and OM

Following MsbA dependent translocation [40,41] the mature LPS molecule assembled at the periplasmic face of the IM by WaaL ligase must traverse the aqueous periplasmic compartment before being inserted and correctly assembled at the OM.

**Figure 2:**
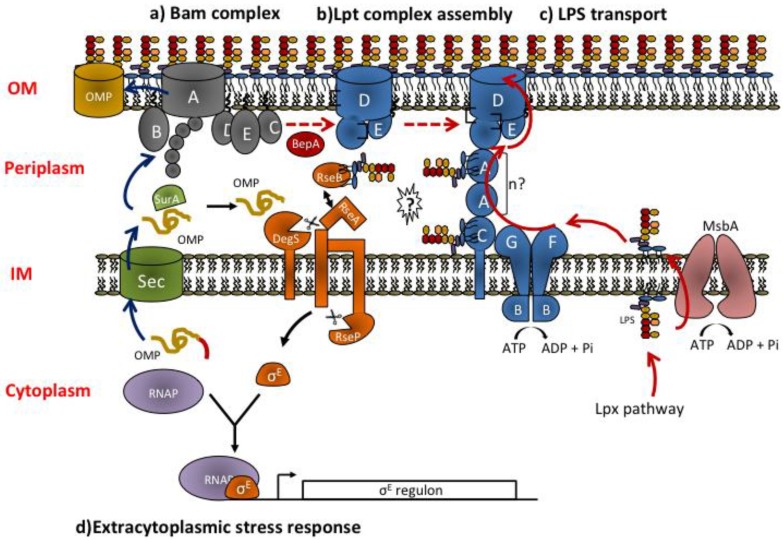
Overview of OMPs and LPS biogenesis pathways and extracytoplasmic stress response. (**a**) OMPs are synthesized in the cytoplasm and translocated across the IM by the Sec translocon. After translocation, the signal sequence (indicated in red) is cleaved. In the periplasm, chaperone proteins as SurA assist OMPs folding and deliver them to the BamABCDE complex for assembly at the OM. Blue arrows show the sequence of events occurring during OMPs biogenesis. (**b**) LptD is an OMP and follows the chaperone/Bam folding pathway. Correct LptD folding requires its association with the lipoprotein LptE and interdomain disulfide bridges isomerization. LptD-LptE interaction at the Bam complex is favored by the chaperone/protease BepA. Red dashed arrows show the sequence of events occurring during LptDE complex assembly. (**c**) LptDE complex is the LPS OM translocon. LPS is synthesized in the cytoplasm by the Lpx pathway, flipped to the periplasmic face of the IM by MsbA and transported through the periplasm to the outer leaflet of the OM by the Lpt machinery. Continuous red arrows show the sequence of events occurring during LPS biogenesis. (**d**) Mislocalized or misfolded OMPs and defects in the LPS export pathway trigger the σ^E^ envelope stress response (black arrows). Misassembled porins bind to DegS, cleaving RseA and initiating the σ^E^ stress response. The current model suggests that defects in Lpt assembly/function result in RseB binding to LPS possibly released by the Lpt machinery (question mark). RseB bound to LPS frees RseA that can then be cleaved by OMP-activated DegS and by RseP thus activating the σ^E^ stress response.

The transport of LPS across the periplasm has been studied mainly in *E. coli* and *N.* *meningitidis*. In *E. coli* the Lpt (lipopolysaccharide transport) complex is composed of seven essential proteins (LptABCDEFG) [53,54,55,56] that are located in every cellular compartment: cytoplasm, IM, periplasm, and OM (Figure 2). The Lpt complex provides energy for LPS extraction from the IM and mediates its transport across the aqueous periplasm and its insertion and assembly at the OM [3,57,58].

The Lpt machinery may be divided in three subassemblies: LptBFGC, LptA, and LptDE, which are located at the IM, in the periplasm, and at the OM, respectively. LptBFG [56,59,60] constitute an IM ABC transporter that is associated to an atypical subunit, the bitopic IM protein LptC [61], whose function in the ABC transporter has not yet been clarified [60]. LptB is the ATP binding domain of this transporter [62] and LptF and LptG represent the transmembrane subunits. At the OM the LptDE translocon composed by the β-barrel protein LptD and the lipoprotein LptE is responsible of the final stage of LPS assembly at the cell surface [55,63]. The periplasmic protein LptA has the role of connecting the two sub-complexes, somehow coordinating their functions [59,64].

The Lpt machinery appears to work as a singe device as depletion of any components leads to similar phenotypes namely block of LPS transport and its accumulation to the periplamic side of the IM where it is decorated by colanic acid by the WaaL ligase [56,61]; such LPS modification appears to be diagnostic of defects in the LPS export pathway [57]. Indeed all the seven proteins physically interact and form a transenvelope complex spanning IM and OM [65].

### 3.2. Molecular Mechanism of Lpt Transport: Towards the Transenvelope Bridge Model

LPS is an amphipatic molecule, this implies that its transport across the periplasm cannot occurs by simple diffusion and requires at least two energy inputs: one for its extraction from the lipid environment of the IM, and one to facilitate the transit of the hydrophobic lipid A portion through the aqueous environment of the periplasm. Since the discovery of the first Lpt factors, this notion induced researchers to postulate different working models to describe the mechanism of LPS transport.

Based on the fact that every envelope compartment has at least one factor dedicated to LPS transport [56,61], two main models have been considered: the chaperone-mediated transit across the periplasm and the transport through a transenvelope proteinaceous bridge spanning both IM and OM.

According to the first model, LptA would be the soluble carrier that accepts LPS from LptBFGC, forms a soluble complex shielding its hydrophobic moiety during the diffusion across the periplasm and, ultimately, delivers it to the OM complex LptDE. As such, LptA would function in the same fashion of LolA, the periplasmic chaperone involved in OM lipoprotein trafficking (see above) [20]. By analogy with LolA, the three-dimensional structure of LptA reveals a novel fold resembling a semiclosed β-jellyroll with an internal hydrophobic cavity [66], compatible with a role in hosting and shielding hydrophobic molecule portions. In fact, LptA binds LPS *in vitro* and *in vivo* [67,68] and can displace it from LptC, in line with their subcellular locations and the unidirectionality of the transport (from IM to OM) [67,68,69]. However, alongside the analogies there are also some substantial differences between Lol and Lpt system: first of all, a recent work from Kahne’s group demonstrated that, unlike LolA, physiologically expressed LptA is not a soluble periplasmic protein but fractionates with both IM and OM in sucrose density gradient centrifugation [65]. Moreover, *in vitro*, LptA shows the propensity to form oligomeric fibrils [66,70]. LptA association to both IM and OM thus suggests that *in vivo* the protein does not function as a soluble carrier but form oligomeric structures spanning the width of the periplasm. This is in agreement with previous evidence that LPS transport occurs in spheroplast, where the periplasmic soluble content has been drained, and that soluble LPS-protein complexes have never been isolated from periplasm [71]. Another remarkable difference between Lol and Lpt system lies in the atypical LptC subunit of the ABC transporter LptBFG. LptC is a bitopic LPS binding protein whose three-dimensional structure closely resembles the LptA fold, despite the low sequence similarity between the two proteins [69]. LptC stably associates to the LptBFG complex, but *in vitro* does not affect the ATPase activity of the transporter [60], making obscure its role in LPS export. Overall, the balance between differences and analogies with the Lol mechanism of transport makes the chaperone-assisted LPS export model unlikely.

The second model for LPS transport suggests the existence of a molecular machine made up of individual protein components located in each cellular compartment thus connecting IM and OM. These molecular machines are not unusual structures in Gram-negative bacteria. Examples are the efflux pumps, responsible for antibiotic resistance in pathogenic bacteria [72], or the Type III secretion systems, whose function is to inject toxins into the infected host cells [73].

The most important evidence supporting this model is that depletion of any Lpt factor leads to accumulation of *de novo* synthesized LPS at the periplasmic side of the IM [61]. This suggests that the Lpt proteins constitute a molecular machine that operates as a single device downstream of MsbA-mediated LPS flipping across the IM (see above). In line with this model, a recent work from Kahne’s group demonstrated that the Lpt proteins co-fractionate in sucrose density gradient centrifugation in a lighter OM fraction (referred to as OM_L_) containing IM and OM components and that these proteins all physically interact to form a transenvelope bridge [65]. The finding that mutations impairing Lpt complex assembly result in degradation of the periplasmic component LptA [74], confirms the biological relevance of the transenvelope structure *in vivo*.

Interestingly, the transenvelope model of transport is reminiscent of a model postulated more than 40 years ago by Manfred E. Bayer, who based on transmission electron micrographs of plasmolysed cells suggested the existence of lipid bridges connecting IM and OM with the function of facilitating the transit of hydrophobic molecules as LPS across the aqueous periplasmic compartment [75,76]. Although initially controversial, this hypothesis obtained confirmations in two studies published some years later demonstrating the existence of zones of adhesion between IM and OM where newly synthesized LPS transiently appears during cell growth [77]. These structures were then isolated as a lighter OM fraction (the same OM_L_ fraction described later by Kahne and co-workers) containing IM and OM components along with murein [78].

To date, the evidence of the existence of these bridges made up of proteins appears definitive. However, some issues concerning the molecular mechanism of LPS transport mediated by the Lpt system still need to be clarified: first, how do the Lpt proteins physically favor the transit of LPS across the periplasm? And second, is the ATP hydrolysis needed to assemble the bridge or is it only required to extract the LPS from the IM?

Some clues about the mechanism by which the transenvelope protein machine physically favors LPS transit through the periplasm arose from recent work by Kahne’s group exploiting the powerful tool of the photo-crosslinking *in vivo*. This chemical approach allows the identification of protein residues involved in protein-protein or protein-ligand interactions by labeling the proteins of interest *in vivo* with an UV reactive cross-linkable aminoacid analog [79,80].

Using this method the authors demonstrated that LptE, which was previously found to reside within the lumen of the β-barrel of LptD [63], interacts directly with some residues of a predicted extracellular loop of LptD adopting a “plug and barrel” architecture [81]. As LptE also binds LPS *in vitro* [63], this finding suggests a dual role for this protein: a structural component of the LptDE complex and a recognition site for LPS at the OM [63,81]. In a more recent work, the same group identified the regions in LptA, LptC, and in the *N*-terminal domain of LptD that mediate contacts between the proteins and thus are implicated in the formation of the transenvelope bridge. These studies showed that *in vivo* LptA contacts LptC at the IM via its *N*-terminal region and LptD at the OM via its *C*-terminal region thus creating a continuous bridge of antiparallel β-strands between IM and OM [64]. Indeed, LptA and LptC belong to the same OstA superfamily of the *N*-terminal domain of LptD and, as mentioned above, share a very similar three dimensional structure [66,69]. Interestingly, the periplasmic but not the transmembrane domain of LptC appears to be also required for interaction with the IM transporter LptBFG [82]. As the periplasmic loop of LptF and LptG are predicted to assume the β-jellyroll structure shared by LptA, LptC and LptD it has been proposed that the transenvelope bridge is based on the conserved structurally homologous jellyroll domain shared by five out of the seven Lpt components [82]. These observations are in agreement with the genomic evidence that many Gram-negative organisms encode predicted fusion proteins containing as many as four OstA superfamily domains [83]. It is not yet known how many LptA molecules compose the transenvelope bridges. In the work by Freinkman and coworkers, the residues identified for LptA-LptA dimer interaction are the same involved in LptA interaction with LptC or LptD and the authors claim that this could be an artifact due to LptA overexpression [64]. Based on structural data, it has been suggested that four OstA superfamily domains are necessary to span the width of the periplasm [66]: if LptC and LptD periplasmic domains are taken into account, LptA would function as a dimer within the transenvelope bridge. Alternatively, the periplasm could be constricted in the vicinity of Lpt bridges, and thus only one LptA molecule would be sufficient to build the bridge.

Finally, Okuda and co-worker developed a system to dissect LPS transport into different steps, shedding light into the role of ATP hydrolysis during the transport process [68]. In this work, the authors identified different sites inside the β-jellyroll structure of LptA and LptC that are involved in LPS interaction *in vivo*, confirming that the periplasmic transit of LPS occurs inside the hydrophobic groove formed by the oligomeric protein bridge. More interestingly, the authors suggested that LPS transits along Lpt components in an ordered process that requires LptBFG to transfer LPS to LptC and then LptBFGC to deliver LPS to LptA. Remarkably, energy (ATP hydrolysis) is not required for assembly of the bridge, instead two energy dependent steps are necessary to translocate LPS from IM to OM: the first requiring energy step is the extraction of LPS from the IM and the second is the transfer of LPS from LptC to LptA. In conclusion, the current model of LPS transport suggests that ATP hydrolysis is used to push a continuous stream of LPS through the transenvelope Lpt bridge in discrete steps against a concentration gradient [68].

### 3.3. Regulation of Lpt Complex Assembly and LPS Transport Process

As discussed above, LPS is a crucial structural component of the bacterial OM that it is responsible of the permeability barrier proprieties of this membrane [12]. Moreover, this complex glycolipid is also believed to function as a molecular chaperone for OMPs assembly: for example, LPS has an established role in trimerization of OmpC and OmpF porins [84,85,86], and it is required for maintaining their proper channel gating function [87]. Due to the important roles played by LPS at the OM and the dangerous consequences of impaired transport of this molecule to the cell surface (for instance, accumulation of LPS in the IM could influence the proper assembly of essential IM proteins that can be lethal for cells), it is not surprising that the bacteria have evolved a fine tuned quality control system to avoid LPS mistargeting during OM biogenesis. In *E. coli* this system comprises a panoply of chaperones assisting the assembly of the LPS OM translocon (LptDE) and molecular mechanisms to ensure that only functional OM translocons are coupled to active Lpt transenvelope bridges allowing LPS translocation from IM to OM.

LptD is an OM protein with a large periplasmic *N*-terminal domain, which is essential for its function *in vivo* [63], and contains four cysteine residues, two in the *N*-terminal and two in the *C*-terminal domain of the protein [54,88], implicated in the formation of two non consecutive interdomain disulfide bonds. Being an OMP, LptD is synthesized in the cytoplasm with an *N*-terminal cleavable signal peptide that allows its transport across the SEC translocon [89]. After secretion across the IM, the signal peptide-processed LptD is transported along the aqueous and oxidizing environment of the periplasm with the aid of chaperones to be finally delivered to the BAM machinery that inserts it into the OM (see above), [22]. The main chaperone involved in LptD folding is SurA [90]. *∆surA* mutant displays lower LptD level and severe OM perturbations, typical phenotypes of mutants impaired in LPS transport [21]. It has been recently demonstrated that the chaperone Skp, belonging to a secondary periplasmic folding pathway together with the chaperone/protease DegP [21,91], acts in concert with SurA to efficiently assemble LptD. The role performed by Skp is masked when another chaperone protein, FkpA, is present in the cell. Interestingly, Skp has a role different from that played by SurA, as overexpression of SurA cannot restore LptD biogenesis in *∆skp ∆fkpA* double mutants [92]. The unique role of Skp/FkpA in LptD biogenesis seems to be correlated with the presence of the large periplasmic *N*-terminal domain, which is the first to emerge into the periplasm during LptD translocation. This would fit well with the observation that Skp association to OMPs occurs at the IM [93]. Based on these results, it is not surprising that FhuA, a protein with a similar structure of LptD, depends on the same group of chaperones for its folding [92].

However, the complex folding process of LptD must take into account two additional proprieties of the mature LPS OM translocon: the interaction with LptE and the presence of disulfide bonds. First of all, in the mature translocon the lipoprotein LptE resides inside the lumen of the *C*-terminal domain of LptD and this interaction is required for the correct folding of LptD β-barrel into the OM by the Bam complex [81,94]. LptDE complex folding is further challenged by the fact that LptD contains two disulfide bonds whose correct formation is required to build a functional translocon [88]. Periplasmic oxidase DsbA is involved in the formation of disulfide bonds in LptD [95], however the first oxidized folding intermediate of LptD is not active as it presents a disulfide bond between the first two *N*-terminal consecutive cysteines [96]. Interaction with LptE to form the “plug and barrel” architecture triggers the conversion of this non-functional intermediate to mature LptD possessing native disulfide bonds between non-consecutive cysteines. Therefore interaction of LptE with LptD ultimately controls the activation of the LptDE traslocon. Interestingly, LptD folds its β-barrel structure before disulfide bonds rearrangement and this folding is rate-limiting for its maturation [96]. Such non-functional folding intermediate is crucial to avoid LPS mistargeting: LptDE translocon becomes active only if both components are correctly synthesized, targeted and assembled together in the OM. This kind of regulatory mechanism is crucial to coordinate the assembly of a translocon whose components must follow different transport pathways (chaperones/BAM for LptD and Lol for LptE) to reach the final destination at the OM.

Very recently, an additional quality control system that assists LptDE translocon folding/oxidation has been identified: it relies on the chaperone/protease BepA. BepA has been proposed to promote efficient LptD-LptE interaction at the Bam complex, as overexpression of LptE suppresses the delay in LptD disulfide bonds rearrangement observed in *∆bepA* mutant and as BepA itself physically and genetically interacts with BamA [97]. Interestingly, in *∆bepA* mutant the LptD non-functional intermediate with non native disulfide bonds is stabilized and overexpression of protease-dead BepA mutants does not completely rescue LptDE translocon functionality, suggesting that the protease activity of BepA is required to degrade LptD when it fails to be correctly inserted into the OM [97].

There is a strong link between LptDE translocon folding and Lpt transenvelope complex assembly. It has been demonstrated that the *N*-terminal domain of LptD has the essential function of connecting the OM translocon to the transenvelope bridge assembly by interacting with the *C*-terminal region of LptA [64]. However, *in vivo* this interaction can occurs only when at least one native disulfide bond is formed in LptD [64] that ultimately depends on the proper interaction between LptD and LptE [96]. This suggests the existence of a regulatory mechanism where the *C*-terminal β-barrel domain of LptD prevents the *N*-terminal periplasmic domain from interacting with LptA when its plug domain (LptE) is not properly inserted. Accordingly, it has been shown that mutations impairing LptE plug function result in defective LPS transport and, as a consequence, in OM barrier perturbations [98]. The existence of such regulatory mechanism is in line with the observation that upon LptC, LptD, or LptE depletion LptA is degraded and LPS accumulates at the IM [61,74]. Moreover it is known that mutations in LptC impairing interaction with LptBFG fail to recruit LptA and therefore to assemble the Lpt transenvelope complex [82]. Overall these data suggest that the cell cannot tolerate the formation of defective transenvelope bridges or the leakage of LPS molecules within the periplasm.

The importance of mechanisms that monitor the proper delivery of LPS to the cell surface is also outlined by a recently identified dedicated surveillance system that detects directly LPS mistargeting and triggers the envelope stress response [99].

In *E. coli* OM integrity is monitored by an envelope stress response system that relies on activation of the essential alternative σ factor (σ^E^) which controls the expression of genes required for damage-repair pathways [100]. Notably, LptA [101] and LptD [102] belong to the σ^E^ regulon. Under non-stress conditions, σ^E^ is kept inactive at the cytoplasmic site of the IM by the negative regulator RseA through binding to its cytoplasmic domain. RseA is a single-pass IM protein whose periplasmic domain binds the regulatory protein RseB and this binding enhances σ^E^ inhibition [103,104]. When an OM stress occurs, the OMPs cannot be properly folded and accumulate into the periplasm. The *C*-terminal residues of unfolded OMPs interact with the protease DegS activating the cleavage of RseA [105]. Following the proteolytic cleavage of the periplasmic region of RseA by DegS, the multipass IM protease RseP cleaves the transmembrane region of RseA thus freeing σ^E^ [100]. However, RseA cleavage by activated DegS is prevented by RseB binding to RseA [103,104,106]. A recent work by Lima and co-workers demonstrated that the cell senses defects in LPS transport through LPS binding by RseB in the periplasm and that LPS-RseB interaction results in RseA-RseB, dissociation allowing RseA cleavage by DegS with consequent σ^E^ activation [99]. This two-signals regulation appears a strategy adopted by the cell to ensure a precise homeostatic control of the barrier function of the OM and reflects the strict connection between the biogenesis of its different components, as defects in LPS biogenesis are expected to generate problems with OMPs assembly and *vice versa*.

## 4. Conclusions

LPS is an essential hallmark of the OM in the majority of Gram-negative bacteria species. The reason why this complex glycolipid is essential for these bacteria is object of a long-standing debate, as certain genera of Gram-negatives do not require LPS to assemble a functional OM and to survive [107,108]. What is clear is that the asymmetry of the OM, ensured by the presence of LPS in its outer leaflet, is fundamental for the barrier proprieties of the OM that contribute to the generalized resistance of Gram-negative bacteria to several antibiotics and allow Enterobacteria to colonize the gut, where high concentration of detergent-like molecules as bile salts need to be tolerated [12]. LPS is a potent activator of the innate immune response and an important virulence factor, making its maturation and assembly at the OM a problem of high biological relevance.

The work of the past 10 years has allowed the discovery of the transenvelope protein machinery that transports the amphipathic LPS molecules across the periplasm at the expense of ATP hydrolysis. Remarkably, the assembly of this machine is finely regulated to ensure correct and co-ordinated OM synthesis. However, several questions remain to be solved. For instance, it is not clear whether the transenvelope structure is always present in the cell or if it is only transiently built and, in this case, whether the presence of LPS in the outer leaflet of the IM is the signal that triggers bridges formation. Moreover, while the interaction between the OM translocon and LptA has been characterized in detail, the assembly of the IM LptBFGC sub-complex and its interaction with LptA is not fully understood. Additional unsolved problem are the number and arrangements of LptA molecules in the transenvelope bridge, as well as the mechanism of LPS insertion in the OM by LptDE. Finally, the studies on LPS export have been performed using *E. coli* K12 strains, that produce a rough-type LPS [43,44], but it is conceivable that in naturally occurring strains the addition of the *O*-antigen moiety to the core-lipid A would generate a considerable steric hindrance. Is some other unknown protein/protein complex involved to the transport of such bulky molecules within the periplasm? Interestingly, it has been suggested that under specific growth conditions the macrolide transporter MacAB-TolC [109] might be involved in LPS transport to the cell surface, as MacA has been shown to contain two LPS binding sites that are essential for the functionality of the transporter in drug resistance *in vivo* and for ATPase activity stimulation *in vitro* [110]. It would be interesting to investigate whether this or other apparently non-related transporters might in turn become essential for growth in strains producing a smooth-type LPS.

It is clear that we are still far from drawing the complete picture of the molecular mechanisms of LPS transport within bacterial envelope and further studies exploiting innovative strategies are, thus, required.
